# Direct Inkjet Printing of Silver Source/Drain Electrodes on an Amorphous InGaZnO Layer for Thin-Film Transistors

**DOI:** 10.3390/ma10010051

**Published:** 2017-01-10

**Authors:** Honglong Ning, Jianqiu Chen, Zhiqiang Fang, Ruiqiang Tao, Wei Cai, Rihui Yao, Shiben Hu, Zhennan Zhu, Yicong Zhou, Caigui Yang, Junbiao Peng

**Affiliations:** Institute of Polymer Optoelectronic Materials and Devices, State Key Laboratory of Luminescent Materials and Devices, South China University of Technology, Guangzhou 510640, China; ninghl@scut.edu.cn (H.N.); c.jianqiu@mail.scut.edu.cn (J.C.); fangzq1230@126.com (Z.F.); 201510102158@mail.scut.edu.cn (R.T.); c.w01@mail.scut.edu.cn (W.C.); hushiben@foxmail.com (S.H.); zhu.zhennan@mail.scut.edu.cn (Z.Z.); zhou.yicong@mail.scut.edu.cn (Y.Z.); 201520114097@mail.scut.edu.cn (C.Y.)

**Keywords:** thin film transistors, inkjet printing, a-IGZO, silver ink

## Abstract

Printing technologies for thin-film transistors (TFTs) have recently attracted much interest owing to their eco-friendliness, direct patterning, low cost, and roll-to-roll manufacturing processes. Lower production costs could result if electrodes fabricated by vacuum processes could be replaced by inkjet printing. However, poor interfacial contacts and/or serious diffusion between the active layer and the silver electrodes are still problematic for achieving amorphous indium–gallium–zinc–oxide (a-IGZO) TFTs with good electrical performance. In this paper, silver (Ag) source/drain electrodes were directly inkjet-printed on an amorphous a-IGZO layer to fabricate TFTs that exhibited a mobility of 0.29 cm^2^·V^−1^·s^−1^ and an on/off current ratio of over 10^5^. To the best of our knowledge, this is a major improvement for bottom-gate top-contact a-IGZO TFTs with directly printed silver electrodes on a substrate with no pretreatment. This study presents a promising alternative method of fabricating electrodes of a-IGZO TFTs with desirable device performance.

## 1. Introduction

A thin-film transistor is one of the most important parts of an active matrix liquid crystal display (AMLCD) and an active matrix organic light emitting diode (AMOLED) [1,2,3]. In recent years, printing technology for TFTs has attracted a considerable amount of attention because there is no need for a vacuum process and it could enable direct patterning, eco-friendly, and low-cost processes [4,5,6]. Silver (Ag) ink has been investigated as an alternative approach to low-cost, high-conductivity, printable conductors compared with other inks such as poly anilines [7], PEDOT [8], and Au ink [4]. Many studies regarding all-inkjet-printed OTFTs have been reported [9,10,11,12,13,14]. However, TFTs applied to the panel industry must have both a high mobility and an on/off current ratio, of which OTFTs are not capable [9,15,16]. Recently, amorphous indium–gallium–zinc–oxide (a-IGZO) TFTs with copper (Cu) [17], titanium (Ti) [18], and silver (Ag) [19,20] electrodes by vacuum deposition have been widely developed with excellent properties, and various novel device technologies have been reported using IGZO or novel channel materials for flexible transparent electronic applications [3,21,22]. If the vacuum processes of electrodes could be replaced by printing technologies, production costs could be saved.

However, poor interfacial contact and/or organic diffusion between semiconductors and silver electrodes would adversely affect the device performance [4,9]. Yoshihiro et al. reported the direct printing of silver electrodes on a-IGZO, but did not get good TFT characteristics due to the presence of carbon and hydrogen. Ethan et al. found that silver-based inks form poor electrical contact to IGZO due to deleterious interfacial chemical interactions, which results in a poor and unstable electrical operation [23].

In this study, we aimed to fabricate a-IGZO TFTs with desirable device performance by directly printing Ag source/drain (S/D) electrodes on a 400 °C pre-annealed semiconductor layer. Carbon was still detected at the interface between the a-IGZO and the Ag electrodes, but relatively better contact between the electrodes and the semiconductor layer was obtained due to the diffusion of silver into an a-IGZO semiconductor layer, which contributed to desirable device performance.

## 2. Experimental

**Figure 1:**
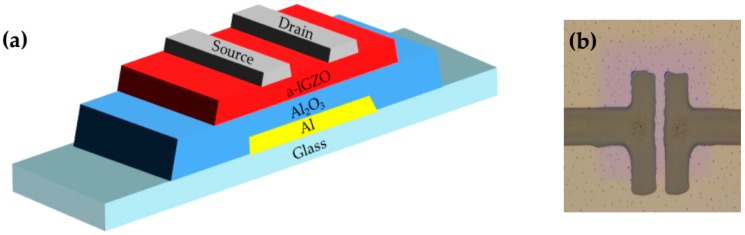
(**a**) Structure of printed S/D electrodes TFT; (**b**) the final device we have fabricated.

The cross-sectional of a-IGZO TFT with inkjet-printed silver S/D electrodes is illustrated in Figure 1a. The fabrication processes are given as follows: firstly, a 300-nm-thick Al gate was deposited on cleaned glass by DC sputtering and patterned by wet etching. Subsequently, the film was anodized in the electrolyte consisting of an ammonium tartrate solution and ethylene glycol. As a result, a 200-nm-thick Al_2_O_3_ insulator gate was formed in an electrolyte consisting of a 3.68 wt % ammonium tartrate solution and ethylene glycol on the Al gate. After that, a 25-nm-thick a-IGZO film patterned via shadow mask was deposited on the insulating layer by RF magnetron sputtering, and the obtained device was annealed at 400 °C for 1 h under atmospheric conditions. At last, a printing process was employed to form the silver source and drain electrodes, which is shown in Figure 1b.

The silver ink (30%–35% in volume) for the electrodes consists of silver nanoparticles (30–50 nm) and alcohol-based solvent (DGP 40TE-20C, Advanced Nano Products, Bugang-myeon, Sejong-si, Korea). The silver S/D electrode was printed onto the a-IGZO layers through an inkjet printer with a 10 pL print head driven by piezoelectricity (Fujifilm Dimatix, DMP2800, Santa Clara, CA, USA). Ag ink was printed using an optimized wave form and cartridge temperature of 30 °C. The droplets were deposited with a dot spacing of 35 μm and resulted in a smaller channel width/length of 551.29 µm/31.09 µm. After printing, UV curing equipment was used to dry the Ag ink with the condition of 100% intensity for 180 s in air.

The dimensions of the printed electrodes were measured by a Nikon Eclipse E600 POL with a DXM1200F digital camera (Nikon, DeWitt, IA, USA). TFT properties were studied using a semiconductor parameter analyzer (Agilent 4155C, Santa Clara, CA, USA) under ambient condition. TEM with an energy dispersive X-ray spectrometer (EDS, Bruker, Adlershof, Berlin, Germany) was used to analyze the distribution of elements, and carbon was detected by EELS (Electron Energy Loss Spectroscopy, Gatan Enfinium ER Model 977, Pleasanton, CA, USA).

## 3. Results and Discussion

**Figure 2:**
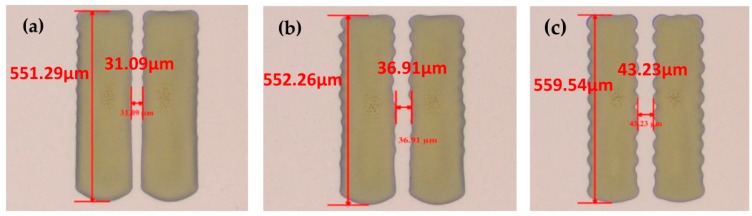
Inkjet-printed Ag S/D electrodes based on Al:Nd/Al2O3:Nd/a-IGZO with different drop spaces: (**a**) 35 µm; (**b**) 40 µm; (**c**) 45 µm.

The silver S/D electrodes with different dot spacing were inkjet-printed on the a-IGZO layer by controlling the droplet spacing. As we can see from Figure 2, the length of the channel increased with the increase in dot spacing. The wavy edge became distinct as the drop space increased. In order to avoid the influence of the edges and obtain a smaller channel length, 35 µm dot spacing was set.

**Figure 3:**
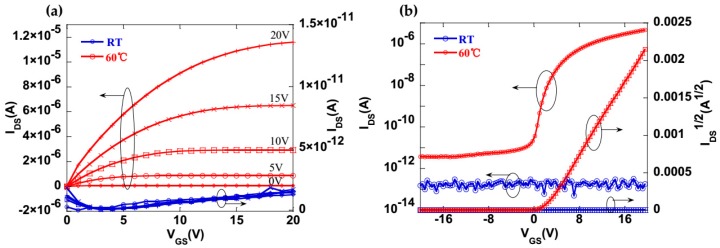
Output characteristic curves (I_DS_–V_DS_) (**a**) and transfer characteristic curves (I_DS_–V_GS_) (**b**) of manufactured a-IGZO TFTs with inkjet-printed Ag S/D electrodes as a function of substrate temperatures. V_GS_ is varied from 20 to −20 V with V_DS_ = 10.1 V.

At first, a-IGZO TFTs with printed S/D electrodes were manufactured at room temperature. Figure 3a,b show output characteristic curves (I_DS_–V_DS_) and transfer characteristic curves (I_DS_–V_GS_), respectively. In our work, the gate voltage (V_GS_) was changed from 0 to 20 V in steps of 5 V. The transfer curves were tested with a V_DS_ of 10.1 V and a V_GS_ from 20 to −20 V. A low source/drain current was indicated in the I_DS_–V_DS_ and I_DS_–V_GS_ curves. We suggested the existence of poor interfacial contact and/or serious diffusion between the active layer and the silver electrodes because of the organic solvent. It is quite important to reduce the carbon concentration in the solvent, as reported in [24]. Therefore, we decided to increase the printing substrate temperature. With the increase in substrate temperature up to 50 °C, no obvious improvement on the TFT properties was observed (see in Figure S1), but the TFT exhibits excellent output characteristics in the linear regime from Figure 3a at an equipment limited substrate temperature of 60 °C, and shows better transfer characteristics than the TFTs printed at lower temperature. It enables a mobility of 0.29 cm^2^·V^−1^·s^−1^ and an on/off current ratio over 10^5^, which is a major improvement for a-IGZO TFTs with printed silver electrodes that was reported so far (see Figures S2 and S3). This result clearly indicates good contact between the IGZO and the Ag.

**Figure 4:**
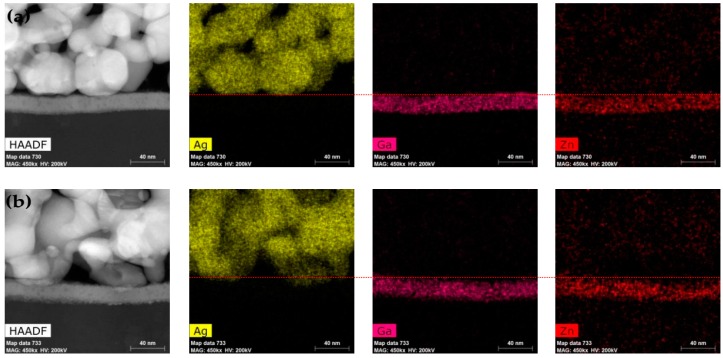
EDS mapping of Ag printed S/D electrodes TFTs with substrate temperature at (**a**) room temperature and (**b**) 60 °C.

To figure out the mechanism that leads to different TFT characteristics, TEM (transmission electron microscope) with an energy dispersive X-ray spectrometer (EDS) was used to observe the interface and detect the distribution of elements after the sample was prepared by a FIB (focused ion beam, FEI Helios 450S dual beam FIB, Milpitas, CA, USA). A clear morphology of Ag particles when printing at room temperature can be observed in Figure 4a. Nanoparticles start melting at a low temperature due to its high surface energy and nanoscale [25]. Therefore, the cross-sectional images in Figure 4 demonstrate the Ag particles merged together at a substrate temperature of 60 °C. As we know, Ag nanoparticles are dispersed by a dispersant, which means the particles are surrounded with organics [26,27]. From Figure 4b, we can see clearly that Ag particles diffused into a-IGZO layers, which contributes to a good contact that finally results in better TFT characteristics.

**Figure 5:**
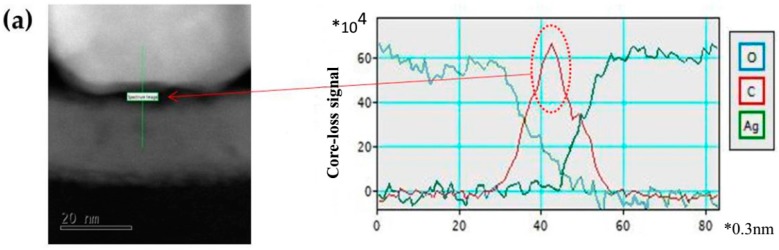

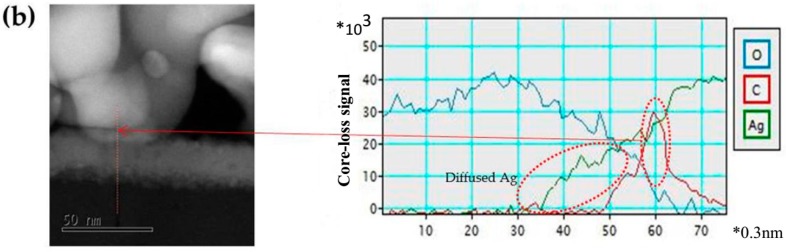
EELS line scanning of a-IGZO/Ag interfaces with printed substrate temperature at (**a**) room temperature and (**b**) 60 °C; the x axis step size of the right pictures is 0.3 nm.

As shown in Figure 5a, a considerable amount of carbon was detected at the interface between Ag and a-IGZO, which reveals the existence of an organic layer that blocks the transportation of electrons. However, for the Ag electrodes printed on a-IGZO with a temperature of 60 °C, no carbon was observed at the interface (Figure 5b)—carbon was only detected on the surface of the Ag particles. Therefore, we can safely conclude that a thin organic layer at the interface isolates the Ag electrodes from a-IGZO, which leads to poor TFT characteristics when printing at lower temperatures. With the increase of the substrate temperature up to 60 °C, Ag nanoparticles melt, and it is beneficial for Ag to diffuse into a-IGZO. Although a few Ag nanoparticles could diffuse into a-IGZO, desirable TFT characteristics were achieved.

## 4. Conclusions

In summary, we achieved desirable TFT characteristics with inkjet-printed Ag S/D electrodes on an a-IGZO layer by increasing the substrate temperature. According to the FIB-TEM results, carbon was detected at the interface between Ag and the a-IGZO, and there was poor contact when the Ag electrodes were printed on the a-IGZO layer at room temperature. As the substrate temperature increased, however, Ag nanoparticles adjacent to the interface merged together and diffused into a-IGZO, resulting in a better contact at the interface. As a result, the device exhibits a mobility of 0.29 cm^2^·V^−1^·s^−1^ and an on/off current ratio of over 10^5^. This study proves the possibility of directly inkjet printing silver electrodes on a-IGZO layer to fabricate TFTs with desirable device performance.

## Supplementary Materials

The following are available online at www.mdpi.com/1996-1944/10/1/51/s1. Figure S1: Output characteristic curves (I_DS_–V_DS_) and transfer characteristic curve (I_DS_–V_GS_) of manufactured a-IGZO TFTs at different printing substrate temperatures. (a) 40 °C; (b) 50 °C; (c) 40 °C; (d) 50 °C. VGS is varied from 20 to −20 V with VDS = 10.1 V, Figure S2: Transfer characteristic curve (I_DS_–V_GS_) of devices Yoshihiro et al. had reported, Figure S3: Transfer characteristic curve (I_DS_–V_GS_) of devices Ethan et al. had reported.
