# Supplementary material for: Direct Inkjet Printing of Silver Source/Drain Electrodes on an Amorphous InGaZnO Layer for Thin-Film Transistors
Source: Materials (Basel). 2017 Jan 10;10(1):51. doi: 10.3390/ma10010051 (PMC5344586; doi:10.3390/ma10010051)
Supplement: Supplementary file 1 [file materials-10-00051-s001.pdf]

Supplementary Materials: Direct Inkjet Printing of Silver Source/Drain Electrodes on an Amorphous InGaZnO Layer for Thin-Film Transistors

Honglong Ning, Jianqiu Chen, Zhiqiang Fang, Ruiqiang Tao, Wei Cai, Rihui Yao, Shiben Hu, Zhennan Zhu, Yicong Zhou, Caigui Yang and Junbiao Peng

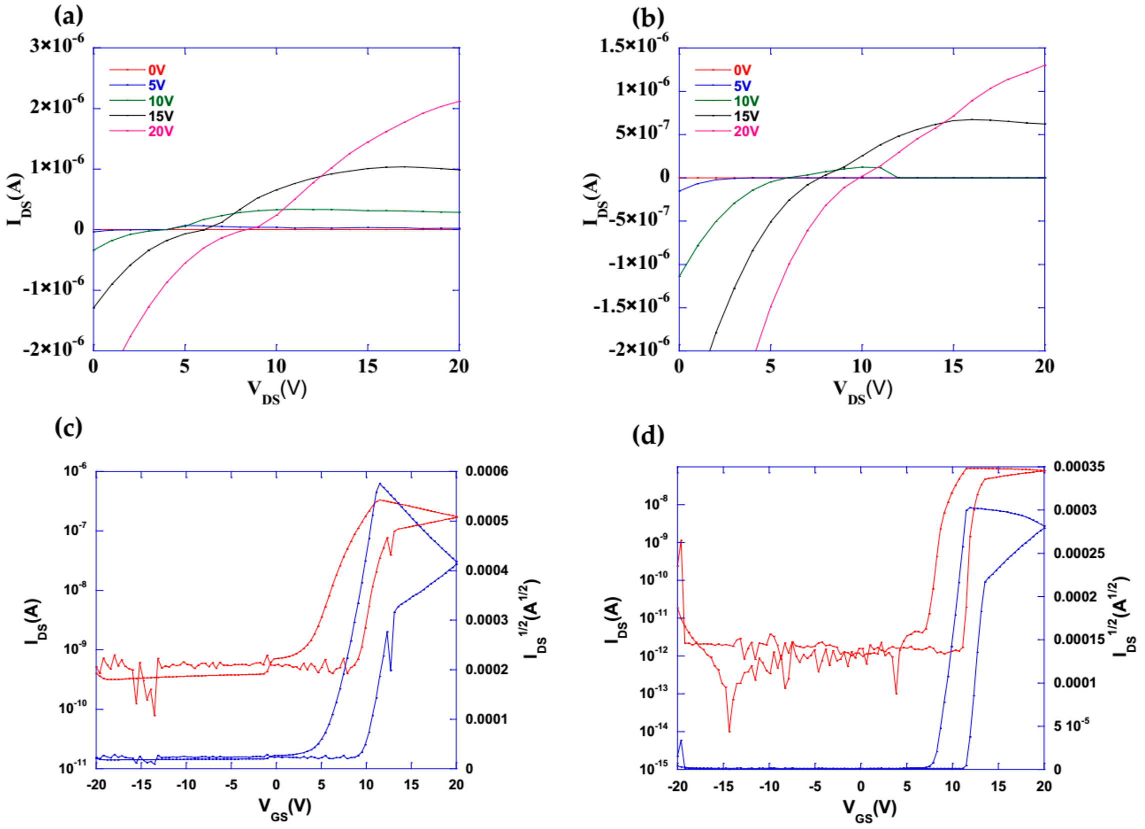

Figure S1. Output characteristic curves (I_{DS}–V_{DS}) and transfer characteristic curve (I_{DS}–V_{GS}) of manufactured a-IGZO TFTs at different printing substrate temperatures. (**a**) 40 °C; (**b**) 50 °C; (**c**) 40 °C; (**d**) 50 °C. V_{GS} is varied from 20 to −20 V with V_{DS} = 10.1 V.

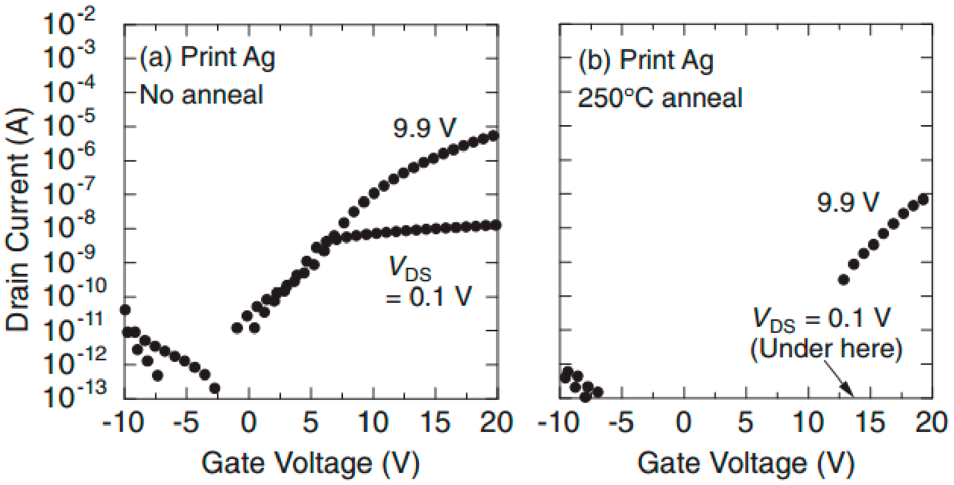

Figure S2. Transfer characteristic curve (I_{DS}–V_{GS}) of devices Yoshihiro et al. had reported.

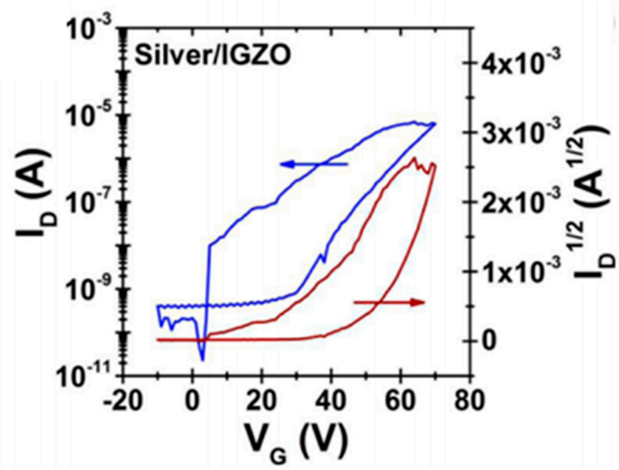

Figure S3. Transfer characteristic curve (I_{DS}–V_{GS}) of devices Ethan et al. had reported.
